# Telephone and face to face methods of assessment of veteran's community reintegration yield equivalent results

**DOI:** 10.1186/1471-2288-11-98

**Published:** 2011-06-25

**Authors:** Linda J Resnik, Melissa A Clark, Matthew Borgia

**Affiliations:** 1 Research Service, Department of Veterans Affairs, 830 Chalkstone Avenue, Providence VA Medical Center, Providence, RI 02908, USA; 2 Health Services, Policy and Practice Section, Department of Community Health, Brown University, 121 South Main Street, 6th floor, Providence, RI 02912, USA; 3 Epidemiology Section, Department of Community Health, Brown University, 121 South Main Street, 6th floor, Providence, RI 02912, USA; 4 Biostatistics Section, Department of Community Health, Brown University, 121 South Main Street, 6th floor, Providence, RI 02912, USA

## Abstract

**Background:**

The Community Reintegration of Service Members (CRIS) is a new measure of community reintegration developed to measure veteran's participation in life roles. It consists of three sub-scales: Extent of Participation (Extent), Perceived Limitations with Participation (Perceived), and Satisfaction with Participation (Satisfaction). Testing of the CRIS measure to date has utilized in-person administration. Administration of the CRIS measure by telephone, if equivalent to in-person administration, would be desirable to lower cost and decrease administrative burden. The purpose of this study was to test the equivalence of telephone and in-person mode of CRIS administration.

**Methods:**

A convenience sample of 102 subjects (76% male, 24% female, age mean = 49 years, standard deviation = 8.3) were randomly assigned to received either telephone interview at Visit 1 and in-person interview at Visit 2, or in-person interview at Visit 1 and telephone interview a Visit 2. Both Visits were conducted within one week. Intraclass correlation coefficients, ICC (2,1), were used to evaluate correspondence between modes for both item scores and summary scores. ANOVAs with mode order as a covariate were used to test for presence of an ordering effect.

**Results:**

ICCs (95%CI) for the subscales were 0.92 (0.88-0.94) for Extent, 0.85 (0.80-0.90) for Perceived, and 0.89 (0.84-0.93) for Satisfaction. No ordering effect was observed.

**Conclusion:**

Telephone administration of the CRIS measure yielded equivalent results to in-person administration. Telephone administration of the CRIS may enable lower costs of administration and greater adoption.

## Background

More than 2 million U.S. troops have been deployed in recent conflicts in Iraq and Afghanistan (Operation Enduring Freedom/Operation Iraqi Freedom/[OEF/OIF]). The toll of these wars is high, with 31,800 troops wounded (as of May 2010)[1] and an expected 790,000 expected to seek disability benefits for service related health problems[2]. Returning service members have been reported to face a wide range of problems in returning to community life including psychological problems, mild traumatic brain injury, marital and financial difficulty, problems with alcohol or substance abuse, and motor vehicle accidents [2-5].

A recent survey found that more than half (52%) of OEF/OIF Veterans had problems controlling anger, 49% reported that their participation in community activities had been impacted, and 42% reported problems getting along with an intimate partner [6]. A quarter of returning Veterans reported problems in employment and almost as many (20%) reported legal problems[6].

It is a Department of Veterans Affairs (VA) priority to help these OEF/OIF Veterans return to full participation in community life roles. Thus, measurement of community reintegration is needed to track Veteran health and social functioning and assess the impact of treatment and policy. The Community Reintegration of Service Members (CRIS) is a new measure of community reintegration developed with VA funding to measure participation in life roles as defined by the International Classification of Health and Functioning (ICF)[7].

Items on the CRIS cover 9 aspects, called chapters in the taxonomy of Activities and Participation as described by the ICF: (1) Learning and Applying Knowledge, (2) General Tasks and Demands, (3) Communication, (4) Mobility, (5) Self-care, (6) Domestic Life, (7) Interpersonal Relationships, (8) Major Life Areas, and (9) Community, Social and Civic Life. The CRIS's three scales measure three dimensions: (1) objective and (2) subjective aspects of participation as well as (3) satisfaction with participation. Items from the CRIS measure are shown in Additional File 1, Appendix A. The Extent of Participation scale asks the respondent to indicate how often he or she experiences or participates in specific activities. The Perceived Limitations in Participation scale asks the respondent to indicate his or her perceived limitations in participation. Lastly, the Satisfaction with Participation scale asks the respondent to indicate the degree of satisfaction with different aspects of participation. In designing the CRIS fixed form scales, we included only those items that demonstrated intraclass correlation coefficients (ICCs) > 0.6 in our pilot same-mode test-retest reliability studies [7].

Previous research showed that the three fixed form CRIS scales demonstrated strong reliability, conceptual integrity and construct validity[7,8]. These findings suggest that the CRIS measure possesses strong psychometric properties and support its use as a standardized assessment measure for the monitoring of community reintegration outcomes of Veterans and wounded warriors from recent conflicts.

All testing of the CRIS measures prior to this study utilized in person survey administration. However, administration of the CRIS measure by telephone would expand the utility of the CRIS by lowering the cost and decreasing the burden of administration;[9] and therefore, ultimately increasing the likelihood of the measure's adoption. Telephone surveys do not require travel, are not affected by geographic distribution of subjects, and are easily monitored for quality. Thus, they may be a more economical means of conducting interviews[10]. That said, we were concerned, based on the prior literature, that telephone and in-person administration might yield varying results due to: (a) the CRIS's complex response format which could be confusing by telephone administration, [11] (b) cognitive demands of completing the survey by telephone, [12-14] and (c) greater potential for social desirability bias for in-person interviews [15,16]. Previous studies have reported an ordering effect in repeat administration of quality of life measures using telephone versus mail administration [17], and telephone versus web administration, [18] and recommend that mixing of questionnaire modes be avoided when gathering certain types of data [17,19]. Thus, we examined potential ordering effects in our analyses.

No prior studies have examined the effect of interview mode, or the effect of mode ordering on the responses of subjects to questions related to their community reintegration. Thus, the overall purpose of this study was to test the equivalence of mode of survey administration of the CRIS measure. Specifically, we examined concurrent criterion validity of the telephone administration of the CRIS, examined whether patient responses to the CRIS measure varied by mode of survey administration (telephone or in-person); and examined whether or not order of survey mode administration (telephone or in-person) was associated with differences in score means and variances. We hypothesized that 1) CRIS scores derived from the telephone administration would be equivalent to those derived through in-person administration and 2) order of survey mode administration would not influence CRIS scores.

## Methods

### Sample

A convenience sample of 102 subjects from the Providence VA Medical Center (PVAMC) was recruited. The Institutional Review Board of the PVAMC approved the research study.

### Data Collection

Prior to full-scale study implementation, the interview script was modified to facilitate telephone administration and refined based on experiences during pilot testing with 5 subjects. After completion of the pilot testing, prospective subjects who expressed an interest in study participation were scheduled for an in-person visit with a research assistant whose sole function was to recruit, schedule and consent subjects. After the consent was completed, subjects were randomly assigned to one of two groups and scheduled for interviews. The first group was administered the telephone interview in the first session followed by an in-person interview in a second session. The second group was administered the in-person interview in the first session followed by the telephone interview in a second session. The two data collection sessions for each participant took place within one week. To minimize the possibility of social desirability bias in the telephone-first group, all interviews were conducted by a second research assistant who had not been involved in the recruitment, initial scheduling or consent process.

At the first interview, the following basic demographic data were collected: age, self-identified racial group, ethnicity, current employment status, household income, highest level of educational achievement, and marital status (see Table 1 for breakdown of categories). We asked subjects to indicate whether or not they had children or stepchildren and whether or not they were currently living with any children under the age of 18 years old. We also asked subjects to indicate whether they currently or ever had been diagnosed with major depression, Post Traumatic Stress Disorder (PTSD), any other mental health condition, or alcohol or drug abuse.

**Table 1:** Demographics by Randomization Group (N = 102)

	Group 1 In-Person followed by Telephone (n = 50)	Group 2 Telephone Followed by In-Person (n = 52)	ALL (n = 102)
	Mean (SD) Range	Mean (SD) Range	Mean (SD) Range
**CRIS Extent of Limitations**	50.1 (8.1) 28-65	51.3 (7.3) 25-63	50.7 (7.6) 25-65
**CRIS Perceived Limitations**	51.2 (10.0) 26-70	51.2 (8.8) 29-70	51.2 (9.3) 26-70
**CRIS Satisfaction**	51.7 (10.0) 25-70	53.0 (9.4) 24-69	52.4 (9.5) 24-70
**Age**	50.0 (8.6) 24-59	49.3 (8.1) 23-59	49.6 (8.3) 23-59
	**Frequency (%)**	**Frequency (%)**	**Frequency (%)**
**Gender**			
Male	38 (76.0)	36 (69.2)	74 (72.6)
Female	12 (22.6)	16 (30.8)	28 (27.4)
**Race**			
White	31 (62.0)	40 (76.9)	71 (69.6)
Black	6 (12.0)	5 (9.6)	11 (10.8)
Other	7 (14.0)	2 (3.9)	9 (8.4)
Mixed	6 (12.0)	5 (9.6)	11 (10.8)
**Hispanic**	6 (12.0)	3 (5.8)	9 (8.8)
**Has Children**	39 (78.0)	42 (80.8)	81 (79.4)
**Live with children under 18**	12 (28.0)	15 (28.9)	29 (28.4)
**Employment status**			
Unemployed	8 (16.0)	14 (26.9)	22 (21.6)
Not working due to disability	18 (36.0)	18 (34.6)	36 (35.3)
Work Training	3 (6.0)	3 (5.8)	6 (5.9)
Working part-time	5 (10.0)	5 (9.6)	10 (9.8)
Working full-time	12 (24.0)	7 (13.5)	19 (18.6)
Retired, not working	3 (6.0)	3 (5.8)	6 (5.9)
Retired and Working	1 (2.0)	2 (3.8)	3 (2.9)
**Income**			
No Income	1 (2.0)	4 (7.7)	5 (4.9)
Less than 15K	9 (18.0)	15 (28.9)	24 (23.5)
15K to 25K	12 (24.0)	8 (15.4)	20 (19.6)
25K to 35K	3 (6.0)	6 (11.5)	9 (8.8)
35k to 50k	7 (14.0)	10 (19.2)	17 (16.7)
50K to 75K	7 (14.0)	6 (11.5)	13 (12.8)
Over 75K	11 (22.0)	3 (5.8)	14 (13.7)
**Marital Status**			
Unmarried	12 (24.0)	11 (21.2)	23 (22.6)
Married	20 (40.0)	16 (30.8)	36 (35.3)
Divorced	1 (2.0)	5 (9.6)	6 (5.9)
Separated	16 (32.0)	20 (38.5)	36 (35.3)
Widowed	1 (2.0)	0 (0.0)	1 (1.0)
**Depression Diagnosis**	26 (53.1)	27 (55.1)	53 (54.1)
**PTSD Diagnosis**	19 (40.4)	22 (44.9)	41 (42.7)
**Mental Illness Diagnosis**	13 (26.5)	18 (37.5)	31 (32.0)
**Alcohol/Drug abuse Diagnosis**	29 (58.0)	28 (53.9)	57 (55.9)

### Statistical Methods

We compared characteristics of the two groups: telephone administration first and in-person administration first, using t-tests for continuous variables and chi-square tests for categorical variables. We used intraclass correlation coefficients, ICC (2,1), to evaluate correspondence between modes for both item scores and summary scores. We used the Shrout & Fleiss (type 2,1) intraclass correlation coefficient, a two-way random effects single measure reliability, where the target and the number of measurements on each target are random effects, and the unit of analysis is the individual measurement instead of the mean of measurements[20]. ICCs above 0.5 were considered as an indication of moderate consistency between modes. Items with ICCs lower than 0.5 were inspected for content. Box plots of mean score difference between mode, stratified by type of first interview mode (telephone or in-person), were used to visually display possible modal or ordering effect. Finally, ANOVAs on summary scores with mode order as a covariate were used as a statistical test for presence of any ordering effect.

## Results

### Descriptives

One hundred and two subjects were randomized into two groups. Subjects in group 1 were administered the CRIS measure in-person at Visit 1 and by telephone at Visit 2, and subjects in Group 2 were administered the CRIS measure by telephone at Visit 1 and in-person at Visit 2. Table 1 shows the characteristics of the subjects by group. No statistically significant differences between groups were observed for any of the characteristics shown in Table 1.

### ICC Analyses

Mean, standard deviation and ICC for each of the three CRIS scores are shown in Table 2. ICCs ranged from 0.85 for Perceived Limitations to 0.92 for Extent of Participation. There were three items in the Extent of Participation scale, six items in the Perceived Limitations scale, and one item in the Satisfaction with Participation scale with ICCs below 0.5 (Table 3) Summary scores were equivalent by mode and that there was no evidence of an ordering effect (Table 4).

**Table 2:** Consistency of CRIS Scale Scores by Mode of Administration (Telephone and In-person)

	In-Person	Telephone	
CRIS Scale	Mean (sd)	Mean (sd)	ICC (2,1) (95% CI)
Extent of Participation	5.1 (0.8)	5.1 (0.8)	0.915 (0.876-0.942)
Perceived Limitations	5.2 (1.0)	5.1 (0.9)	0.853 (0.789-0.898)
Satisfaction with Participation	5.3 (1.0)	5.3 (1.0)	0.891 (0.842-0.925)

**Table 3:** Items in CRIS Scales with ICCs below 0

CRIS SCALE	Question	ICC(2,1)	95% CI
EXTENT	How often did you engage in risky behavior?	**0.468**	**0.300, 0.608**
EXTENT	How often were you able to do several things in a row, such as following directions or doing several tasks one after the other?	**0.484**	**0.316, 0.623**
EXTENT	How often did you fulfill all of the duties of your job?	**0.237**	**-0.090, 0.518**
PERCEIVED	I remembered what I read.	**0.433**	**0.259, 0.579**
PERCEIVED	I got along with people at work.	**0.390**	**0.080, 0.631**
PERCEIVED	I was limited in following directions.	**0.482**	**0.316, 0.619**
PERCEIVED	I was limited in keeping track of my daily tasks and activities.	**0.494**	**0.330, 0.629**
PERCEIVED	Others expressed distress while being a passenger in my car.	**0.482**	**0.301, 0.630**
PERCEIVED	I was limited in doing volunteer activities.	**0.422**	**0.245, 0.571**
SATISFACTION	How satisfied were you with your job performance?	**0.466**	**0.171, 0.684**

**Table 4:** Results of ANOVAs of summary scores examining differences between mode of administration and order of interview mode

	Extent Score		Perceived Score		Satisfaction Score	
	F	P	F	P	F	P
Mode	2.49	0.1227	0.50	0.4817	1.67	0.1975
Order	0.64	0.4241	0.12	0.7310	0.03	0.8532
Order × Mode	1.31	0.2534	0.06	0.8020	0.33	0.5664

## Discussion

This study tested the comparability of telephone and in-person modes of administration of a new measure of community reintegration for veterans, called the CRIS. We found, based upon ICCs ranging from 0.85 to 0.92, that summary scores for the three CRIS subscales were largely comparable between modes. The cut-point for acceptable reliability coefficients varies by field of study, with separate values acceptable for different applications. Generally, speaking ICCs above 0.85 are considered acceptable to make decisions about individuals [21]. Nunnally recommends a minimum reliability of 0.70 for use of a scale in research and 0.90 for use in clinical practice [22]. As a point of reference, only two of the widely used scales of the SF-36 have reliabilities above 0.90 [23].

To confirm that our sample size of 102 persons was adequate, we conducted post-hoc power calculations. For the reliability analysis, we estimate that we have achieved power of 80% to detect an ICC of 0.9 under the alternative hypothesis (which is the approximate value for CRIS subscale ICCs), when the ICC under the null hypothesis is 0.81, using an F-test with alpha = 0.05, and two samples of 50 persons each [25].

We found that 141/151 (93%) of items had ICCs of 0.5 or above, indicating moderate reliability at the item level. However, we did note that 10 of 151 CRIS items (< 7%) had ICCs below 0.5, indicating potential non-equivalence of telephone and in-person administration modes for these items. These items included ones about working, risk taking, and multitasking. These findings should be interpreted cautiously because confidence intervals for the ICC estimates in the current study were wide, and the higher bound of the confidence limits for all items exceeded 0.5. Three items with ICC point values below 0.5 were questions about participation in work or work situations. We believe that these items had very large confidence intervals due to the low percentage of respondents who were working (37%) and the smaller number of subjects who answered each of these questions.

The CRIS scales utilize a complex response format consisting of 7-point Likert-like response scales. There are multiple types of response scales in the measure, each with differing categories of responses (See Additional File 2, Appendix B for response scales). Prior research on telephone versus in-person administration reports both advantages and disadvantages of each mode as well as equivalence between modes. De Vaus suggests that in-person interviews may be preferable for surveys of complex questions with multiple response categories because telephone respondents may have difficulty remembering multiple categories when they answer questions with a large number of response categories[11]. While telephone respondents may have response cards mailed to them in advance of an interview, for practical purposes this is less than optimal because it requires advance planning and assumes that respondents refer to the cards appropriately during the interview. Because of this, we did not mail response cards in this study. In contrast, in-person respondents have a visual aid, in the form of the response scale displayed in front of them as they answer each item, as well as an interviewer who can respond to facial expressions suggesting confusion and who can point to the appropriate response display while explaining the item.

Telephone respondents have been reported to be less patient with interviews and to avoid conversation that may lengthen the interview[12]. Some data suggest that telephone interviews are generally completed more quickly than equivalent in-person interviews [13]. Telephone respondents are in an uncontrolled environment, may be distracted during interviews by things in their environment or they may be multi-tasking at home-by watching TV, cooking or even interacting with others while responding to the interviewer. Thus, they may be less likely to exert the mental effort to answer questions carefully[13]. A respondent answering a long survey may lose motivation, become fatigued and/or lose focus and be unable to sustain the mental effort needed to carefully consider and answer survey questions[14]. When these things occur, the respondent may be more likely to respond in a manner that they believe would seem acceptable or reasonable to the interviewer. Non-verbal cues provided through face-to-face interviewing could potentially enhance the motivation of subjects, keeping them more engaged and thus more likely to respond carefully. Furthermore, the more controlled environment of a face-to-face interview can minimize distractions. While we had no way to monitor telephone a respondent's behavior (i.e. potential distractions from multi-tasking), our results suggest that the potential effect on survey responses was negligible.

While in-person respondents may be motivated by the development of greater rapport and enhanced task performance,[15] the presence of an interviewer may create other biases. Face to face interviews may be more biased due to respondents' desire to express socially acceptable characteristics, and may be influenced by the gender and other observable characteristics of the interviewer[11]. Previous research suggests that social desirability bias is more likely to occur when questions relate to sensitive topics such as sexuality, drug use and risk taking behavior; topics that are included in the CRIS [16].

Greater physical distance between the respondent and the interviewer may provide a greater sense of safety and lead to responses that are more candid. Thus, one would expect that face-to-face interviews would diminish social distance and lead to greater social desirability bias in survey responses because the respondent is observed directly by the interviewer who can respond to non-verbal signs of approval, or disapproval in the form of facial expression or body language. This is confirmed by reports that suggest that the greater anonymity associated with telephone surveys yield more candid reports of risky or socially disapproved behavior [25,26]. However others researchers have reported the opposite effect, indicating that respondents to in-person interviews were more likely to report vulnerabilities such as disability, than respondents to telephone interviews [13,27]. It is possible that potential social desirability bias related to sensitive behavior might impact several of the CRIS items, particularly those related to risky behavior and frequency of sexual activities [16].

While it is possible that the lower ICC values of the items related to risk taking behavior and driving safety that we observed in this study might be attributable to social desirability bias, we do not believe that this was the case. If social desirability was a factor, we might expect that subjects would report higher functioning (i.e. higher scores) during the in-person interview as compared to the telephone interview. We would also have expected to find a lower ICC value for the item related to frequency of sexual relations. Our examination of the raw data shows that the mean of the responses to the question, "How often did you engage in risky behavior?" was lower (mean = 6.1, sd = 1.6) for the in-person then it was for the telephone administration (mean = 6.5; sd = 1.2). The mean of the responses to the items, "Others expressed distress while being a passenger in my car," were nearly identical: 5.6 (sd 1.5) for the in person administration and 5.6 (sd 1.4) for the telephone administration. None of these differences were statistically significant. Thus, we believe that the lower ICCs resulted from the wide confidence intervals around the point estimate, rather than differences between modes of administration.

There were five additional items with ICCs below 0.5. Because these items related to multitasking, remembering what was read, keeping track of daily tasks and activities, and limitations in volunteer work we would not have expected them to be particularly affected by social desirability bias. Examination of the raw data (not shown) shows nearly identical means scores for the groups, suggesting that the lower ICC values were not a substantial concern, and reflected a lack of precision around the estimates in this sample. Additional research is necessary to confirm this finding.

Our study design limits inferences about whether or not potential differences in item responses between modes were attributable to the mode of survey administration or to the actual test-retest reliability of the item. Test-retest reliability is not an inherent property of a measurement instrument, but can vary by population[28]. However, prior research using repeat administration of the in-person CRIS in a very similar sample showed that all items had ICCs of > 0.6[7]. Further research testing equivalence of mode of administration is needed to confirm our current findings.

## Conclusion

In conclusion, there appears to be good potential to use the CRIS fixed form measure by telephone administration. The overall scores were comparable between modes and ICC values for the total scores, and 93% of items indicated acceptable reliability. Since publication of the original article describing CRIS development, the author has received multiple inquiries regarding use of the CRIS measure for research, surveillance and clinical assessment of Veterans. Based upon this research, we believe that use of telephone administration is justified by the overall score equivalence, increased convenience and lower cost of this mode of administration.

## Competing interests

The authors declare that they have no competing interests.

## Authors' contributions

LR obtained funding for this study, conceptualized the design, oversaw the project, oversaw the analyses, and took the lead in writing the manuscript. MC assisted in conceptualizing the study design, interpreting the analytical results, and participated in writing and review of the manuscript. MB participated in data cleaning, data analysis, interpretation of results, writing and review of the manuscript. All authors read and approved the final manuscript.

## Authors' information

Linda Resnik, PT, PhD is a Research Health Scientist at the Providence VA Medical Center and Associate Professor (Research) in the Department of Community Health, Brown University, Providence, RI

Melissa A. Clark, PhD is Associate Professor, Department of Community Health and Obstetrics and Gynecology, Brown University

Matthew Borgia, BS is a graduate student in the Department of Biostatistics, Brown University

## Pre-publication history

The pre-publication history for this paper can be accessed here:

http://www.biomedcentral.com/1471-2288/11/98/prepub

## Supplementary Material

Additional file 1 **Appendix A Items in CRIS Measure**. This file contains the items in each of the CRIS scales. Click here for file

Additional file 2 **Appendix B Response scales in CRIS Measure**. This file shows the response scales used in the CRIS measure. Click here for file
