# Supplementary material for: Telephone and face to face methods of assessment of veteran's community reintegration yield equivalent results
Source: BMC Med Res Methodol. 2011 Jun 25;11:98. doi: 10.1186/1471-2288-11-98 (PMC3146953; doi:10.1186/1471-2288-11-98)
Supplement: Additional file 1 — Appendix A Items in CRIS Measure. This file contains the items in each of the CRIS scales. [file 1471-2288-11-98-S1.DOC]

**Appendix A. Items in CRIS Measure**

**Extent Scale**

1. How often did you have a problem or limitation in driving?
2. How often did others avoid being a passenger while you were driving?
3. How often did you avoid being a passenger in a car?
4. How often have others at work complained about the way you did your job, for example, that you talk too much, or they didn't like the way you behave?
5. How often did you have major conflict with your supervisor?
6. How often did you need to be reminded to eat?
7. How often did you engage in risky behavior?
8. How often did your drinking alcohol or using drugs cause you to have trouble at home?
9. How often did your drinking alcohol or using drugs cause you to have trouble with family or friends?
10. How often did you have major conflict with your spouse or significant other?
11. How often did you get confused in a busy or noisy environment?
12. How often did you have difficulty handling day to day problems?
13. How often did you lack motivation and initiative to start new projects, or take care of day to day tasks or chores?
14. How often were you in contact with your family? When thinking of family, please do not include spouse, significant other, or children.
15. On average, how often did you participate in recreational activities, not including watching TV?
16. How often did you engage in hobbies?
17. How often did you exercise or do light to moderate physical activity, such as walking, for at least 30 minutes?
18. How often were you able to do several things in a row such as following directions, or doing several tasks one after another?
19. How often did you spend quality time with your children?
20. How often did you get together, in person, with friends who are non-veterans?
21. How often did you read or watch the local or world news?
22. How often did you follow current events?
23. How often did you fulfill all of the duties of your job?
24. How often did you understand things that you read?
25. How often did you understand complex reading materials, such as long forms, legal documents, or instruction manuals?
26. How often were you able to do two things at once, such as doing a chore and having a conversation?
27. How often did you engage in sexual relations with your spouse or significant other?
28. How often did you go to crowded places?
29. How often did you help your friends, neighbors or relatives that did not live with you?
30. How often did you have a regular daily routine of eating?
31. How often did you follow the instructions or treatment recommendations of your health care provider?
32. How often did you take care of what you needed to do where you lived?
33. How often did you fulfill your financial responsibilities where you lived?
34. How often did you have a problem concentrating on what you were doing?
35. How often did you need to be reminded of important things you've already been told?
36. How often did you have difficulty handling unexpected problems?
37. How often did you need to be reminded to begin important tasks or activities?
38. How often did you need to be reminded to begin basic everyday tasks or activities?
39. How often did your feelings of anxiety and panic cause problems in your life?
40. How often did you feel that others misunderstood what you were trying to say?
41. How often did you find yourself easily frustrated by things that other people said or did?
42. How often did you lose your temper with other people?
43. How often did conflict with others cause major problems in your life?
44. When speaking with others, how often did you interrupt them inappropriately?
45. How often did you avoid socializing with others?
46. How often did you have a problem in moving around or getting around indoors?
47. How often did you have a problem traveling to places?
48. How often did your lack of organization cause problems in your life, such as financial problems or missed appointments?
49. How often did you have difficulty managing your money such as paying your bills or keeping track of your expenses?

**Perceived Limitations Scale**

1. It was easy to concentrate on what I was doing.
2. I was careful and attentive to detail.
3. I remembered what I read.
4. I was able to understand complex reading materials such as long forms, legal documents, or instruction manuals.
5. I was able to start important tasks and activities without being reminded.
6. I was able to do two things at once such as doing a chore and having a conversation.
7. I was able to complete tasks that I started such as doing a chore.
8. I could cope with life’s ups and downs.
9. I found it easy to show concern, love, and warmth to others I cared about.
10. I settled my own conflicts with others through discussion and compromise.
11. Overall, I took care of what I needed to do where I lived.
12. Overall, I felt that I fulfilled my financial responsibilities where I lived.
13. I woke up when I had to.
14. I had a regular, daily routine of eating.
15. I had the transportation I needed to get where I wanted to go.
16. Getting along with others in my family was important to me.
17. I got along with my spouse or significant other.
18. I got along with my friends.
19. I did my job well.
20. I had no problem getting my work done in my job.
21. I got along with my supervisor.
22. I got along with people at work.
23. I was limited in training for a new job.
24. I felt discriminated against in getting a job.
25. I was easily confused when in a busy or noisy environment.
26. I was limited in following directions.
27. I was limited in handling day to day problems.
28. I was limited in using the phone, e-mail, or mail to contact others.
29. People misunderstood what I was trying to say.
30. I was limited in keeping track of my daily tasks and activities.
31. I was easily frustrated by things that other people said or did.
32. I said critical or hostile things to my friends or loved ones.
33. I felt that I might hit or strike someone.
34. Others felt that I interrupted inappropriately when we were talking.
35. I needed to be reminded to eat.
36. I was limited in doing exercise or light to moderate physical activity, such as walking, for at least 30 minutes.
37. I avoided going to crowded places such as the mall, or community gatherings.
38. I avoided going out alone after dark.
39. In general, I avoided being a passenger in a car.
40. Others expressed distress while being a passenger while I was driving.
41. I had a problem or limitation in driving.
42. I put myself or others in harm’s way while driving.
43. Others felt that I need to cut down on my drinking or drug use.
44. Others felt that my actions put my health and safety at risk.
45. Others felt that I was limited in looking after the needs of my children or step-children.
46. I was limited in experiencing physical intimacy.
47. I had difficulty managing my money either in paying my bills or in keeping track of my expenses.
48. I had financial problems because I was careless with money or didn't pay my bills on time.
49. I was limited in doing volunteer activities.
50. I was limited in going places like going to work, going out to a store, or for a walk.
51. I was limited in doing my hobbies.
52. I was limited in participating in recreational activities, not including watching TV.
53. I was limited in engaging in social gatherings.
54. I felt I spent too much time alone.

**Satisfaction Scale**

1. How satisfied were you with your ability to learn new things?
2. How satisfied were you with your ability to start basic everyday tasks and activities without being reminded?
3. How satisfied were you with your relationship with your spouse or significant other?
4. How satisfied were you with your ability to think clearly and logically?
5. How satisfied were you with your ability to think clearly while in a busy or noisy environment?
6. How satisfied were you with your ability to make decisions?
7. How satisfied were you with your ability to handle day to day problems?
8. How satisfied were you with your ability to read long documents or books?
9. How satisfied were you with your ability to understand material you have read?
10. How satisfied were you with your ability to do two things at once such as doing a chore and having a conversation?
11. How satisfied were you with your ability to do several things in a row such as following directions, or doing several tasks one after another?
12. How satisfied were you with your ability to keep track of your daily tasks and activities?
13. How satisfied were you with your ability to get and stay organized?
14. How satisfied were you with the way you coped with life's ups and downs?
15. How satisfied were you with the way that you participated in conversations?
16. How satisfied were you with your ability to make yourself understood?
17. How satisfied were you with moving around or getting around indoors as you wanted to?
18. How satisfied were you with the way you protected yourself from harm?
19. How satisfied were you with the way you managed your stress level?
20. How satisfied were you with the way that you took care of your health?
21. How satisfied were you with your ability to prepare meals?
22. How satisfied were you with your personal cleanliness?
23. How satisfied were you with your participation in exercise or light to moderate physical activity such as walking?
24. How satisfied were you with your ability to control your intake of alcohol or use of drugs (*other than what has been prescribed for you*)?
25. How satisfied were you with your stress level while being a passenger in a car?
26. How satisfied were you with your stress level while driving a car?
27. How satisfied were you with how you took care of what you needed to do where you lived?
28. How satisfied were you with the way you assisted others who lived with you?
29. How satisfied were you with the way you got along with your family? When thinking of family, please do not include spouse, significant other, or children.
30. How satisfied were you with the way you got along with people other than family?
31. How satisfied were you with your ability to control your temper?
32. How satisfied were you with your awareness of what other people were feeling?
33. How satisfied were you with the way you got along with other people?
34. How satisfied were you with the way you acted with friends and loved ones?
35. How satisfied were you with the way you handled major conflicts with others?
36. How satisfied were you with your relationships with people close to you?
37. How satisfied were you with the amount of time you had with friends?
38. How satisfied were you with the way that you met your children's or step-children's needs?
39. How satisfied were you with your participation in social gatherings?
40. How satisfied were you with your relationship with your supervisor at work?
41. How satisfied were you with your relationships with people at work?
42. How satisfied were you with your level of involvement in hobbies?
43. How satisfied were you with the amount of time you spent in recreational activities not including time spent watching TV?
44. How satisfied were you with the way you kept up with the news?
45. How satisfied were you with the number of hours that you worked?
46. How satisfied were you with your job performance?
47. How satisfied were you with your ability to manage your money by paying bills or by keeping track of your expenses?
48. How satisfied were you with your driving?
