# Supplementary material for: Telephone and face to face methods of assessment of veteran's community reintegration yield equivalent results
Source: BMC Med Res Methodol. 2011 Jun 25;11:98. doi: 10.1186/1471-2288-11-98 (PMC3146953; doi:10.1186/1471-2288-11-98)
Supplement: Additional file 2 — Appendix B Response scales in CRIS Measure. This file shows the response scales used in the CRIS measure. [file 1471-2288-11-98-S2.DOC]

**RESPONSE SCALES FOR CRIS MEASURES**

**In the past two weeks, was it?**

**More than Daily 5 to 6 3 to 4 1 to 2 Less than Never**

**once per day times a week times a week times a week once a week**

**In the past two weeks, was it?**

**Never Less than 1 to 2 3 to 4 5 to 6 Daily More than**

**once a week times a week times a week times a week once per day**

**In the past two weeks, was it?**

**1 2 3 4 5 6 7**

**Not at All Rarely Occasionally Sometimes Often Very often Always**

**In the past two weeks, was it?**

**7 6 5 4 3 2 1**

**Not at All Rarely Occasionally Sometimes Often Very often Always**

**In the past two weeks:**

**1 2 3 4 5 6 7**

**Completely Very much Disagree Mixed Agree Very much Completely**

**Disagree Disagree Agree Agree**

**In the past two weeks:**

**7 6 5 4 3 2 1**

**Completely Very much Disagree Mixed Agree Very much Completely**

**Disagree Disagree Agree Agree**

**In the past two weeks:**

**1 2 3 4 5 6 7**

**Very Unhappy Mostly Mixed Mostly Happy Very Unhappy Dissatisfied Satisfied Happy**
